# Trilateral association of autophagy, mTOR and Alzheimer’s disease: Potential pathway in the development for Alzheimer’s disease therapy

**DOI:** 10.3389/fphar.2022.1094351

**Published:** 2022-12-22

**Authors:** Arunkumar Subramanian, T. Tamilanban, Abdulrhman Alsayari, Gobinath Ramachawolran, Ling Shing Wong, Mahendran Sekar, Siew Hua Gan, Vetriselvan Subramaniyan, Suresh V. Chinni, Nur Najihah Izzati Mat Rani, Nagaraja Suryadevara, Shadma Wahab

**Affiliations:** ^1^ Department of Pharmacology, SRM College of Pharmacy, SRM Institute of Science and Technology, Chengalpattu, Tamilnadu, India; ^2^ Department of Pharmacognosy, College of Pharmacy, King Khalid University, Abha, Saudi Arabia; ^3^ Complementary and Alternative Medicine Unit, King Khalid University, Abha, Saudi Arabia; ^4^ Department of Foundation, RCSI & UCD Malaysia Campus, Georgetown, Pulau Pinang, Malaysia; ^5^ Faculty of Health and Life Sciences, INTI International University, Nilai, Malaysia; ^6^ Department of Pharmaceutical Chemistry, Faculty of Pharmacy and Health Sciences, Royal College of Medicine Perak, Universiti Kuala Lumpur, Ipoh, Perak, Malaysia; ^7^ School of Pharmacy, Monash University Malaysia, Bandar Sunway, Selangor, Malaysia; ^8^ Faculty of Medicine, Bioscience and Nursing, MAHSA University, Bandar Saujana Putra, Selangor, Malaysia; ^9^ Department of Biochemistry, Faculty of Medicine, Bioscience, and Nursing, MAHSA University, Bandar Saujana Putra, Selangor, Malaysia; ^10^ Department of Periodontics, Saveetha Dental College and Hospitals, Saveetha Institute of Medical and Technical Sciences, Chennai, India; ^11^ Faculty of Pharmacy and Health Sciences, Royal College of Medicine Perak, Universiti Kuala Lumpur, Ipoh, Perak, Malaysia

**Keywords:** Alzheimer’s disease, mTOR pathway, dementia, autophagy, tau protein

## Abstract

The primary and considerable weakening event affecting elderly individuals is age-dependent cognitive decline and dementia. Alzheimer’s disease (AD) is the chief cause of progressive dementia, and it is characterized by irreparable loss of cognitive abilities, forming senile plaques having Amyloid Beta (Aβ) aggregates and neurofibrillary tangles with considerable amounts of tau in affected hippocampus and cortex regions of human brains. AD affects millions of people worldwide, and the count is showing an increasing trend. Therefore, it is crucial to understand the underlying mechanisms at molecular levels to generate novel insights into the pathogenesis of AD and other cognitive deficits. A growing body of evidence elicits the regulatory relationship between the mammalian target of rapamycin (mTOR) signaling pathway and AD. In addition, the role of autophagy, a systematic degradation, and recycling of cellular components like accumulated proteins and damaged organelles in AD, is also pivotal. The present review describes different mechanisms and signaling regulations highlighting the trilateral association of autophagy, the mTOR pathway, and AD with a description of inhibiting drugs/molecules of mTOR, a strategic target in AD. Downregulation of mTOR signaling triggers autophagy activation, degrading the misfolded proteins and preventing the further accumulation of misfolded proteins that inhibit the progression of AD. Other target mechanisms such as autophagosome maturation, and autophagy-lysosomal pathway, may initiate a faulty autophagy process resulting in senile plaques due to defective lysosomal acidification and alteration in lysosomal pH. Hence, the strong link between mTOR and autophagy can be explored further as a potential mechanism for AD therapy.

## Graphical Abstract

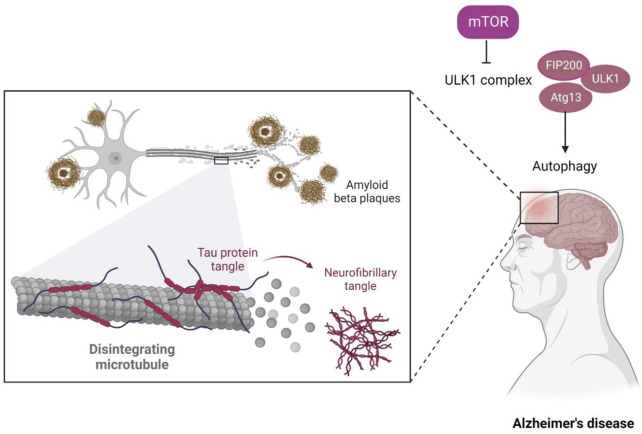

## 1 Introduction

Many individuals above 65 years of age tend to suffer from a general progressive neurodegenerative disorder called Alzheimer’s disease (AD) (Scheltens et al., 2016). The disorder includes the formation of amyloid-β peptide (Aβ) aggregates as a result of proteolytic processing of the amyloid precursor protein (APP) and, to date, has no effective treatment (Oddo et al., 2006). Age is the prime risk factor for the progression of AD, the prevalent type of dementia spreading across the world, where 40 million individuals are affected (Selkoe and Hardy, 2016), and the count is expected to triple by 2050 (Galvan and Hart, 2016).

**FIGURE 1:**
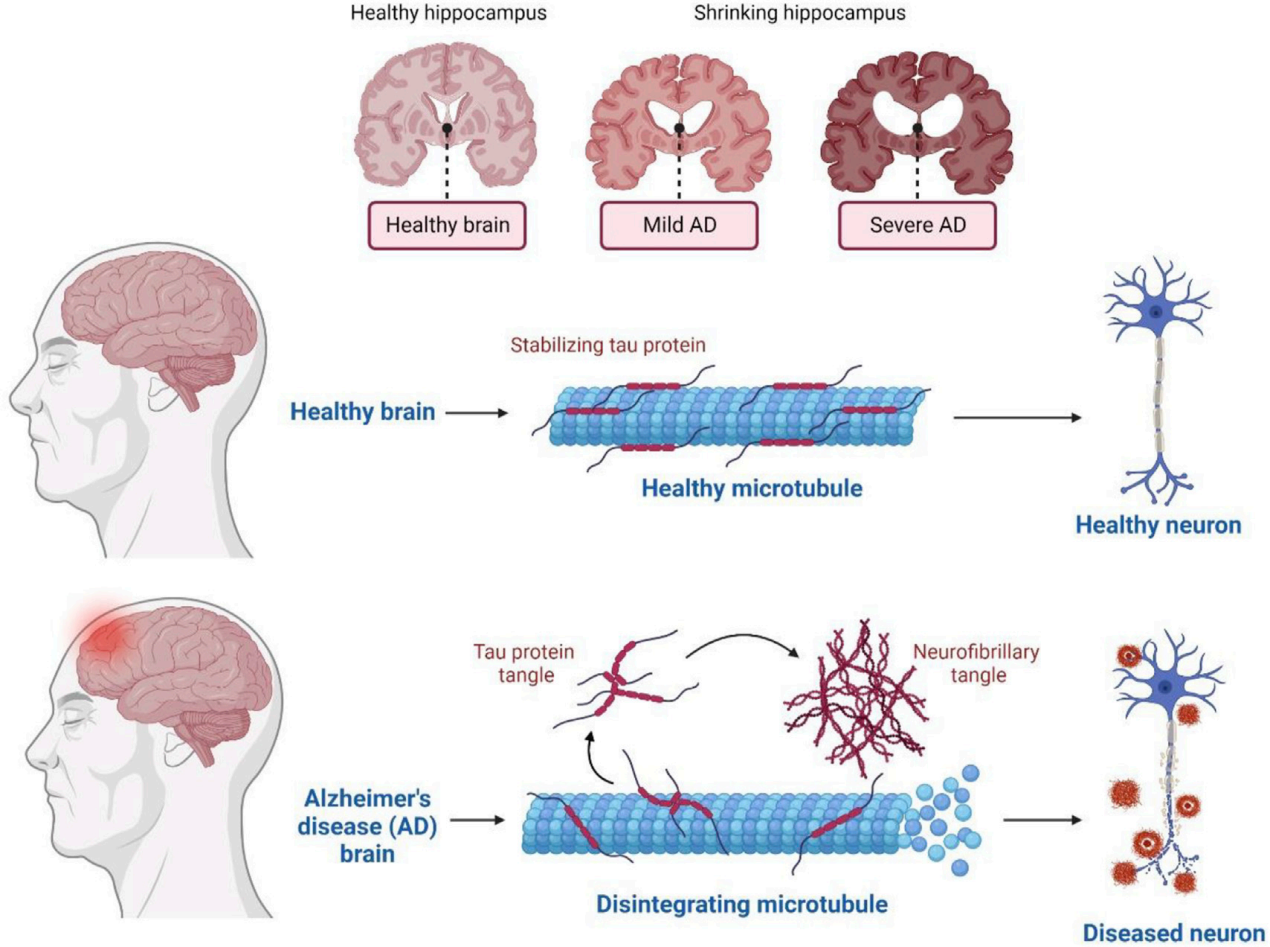
Physiological structure in Healthy and AD human Brain.

In terms of genetics, AD-inherited patients show the presence of a mutated amyloid precursor protein (APP) gene with an autosomal dominant trait and mutated presenilin genes. Clinically, AD is also illustrated by cognitive impairment, overproduction of Aβ aggregates, and tau protein’s hyper-phosphorylation in many basic research studies. AD is a gradually progressive neurodegenerative disease prominently consisting of 1) neuritic senile plaques and neurofibrillary tangles (NFT) (Figure 1) 2) shrinkage of the hippocampus region, resulting in the accumulation of Aβ aggregates in the medial temporal lobe, and 3) neocortical structures of the affected human brain (De-Paula et al., 2012).

**FIGURE 2:**
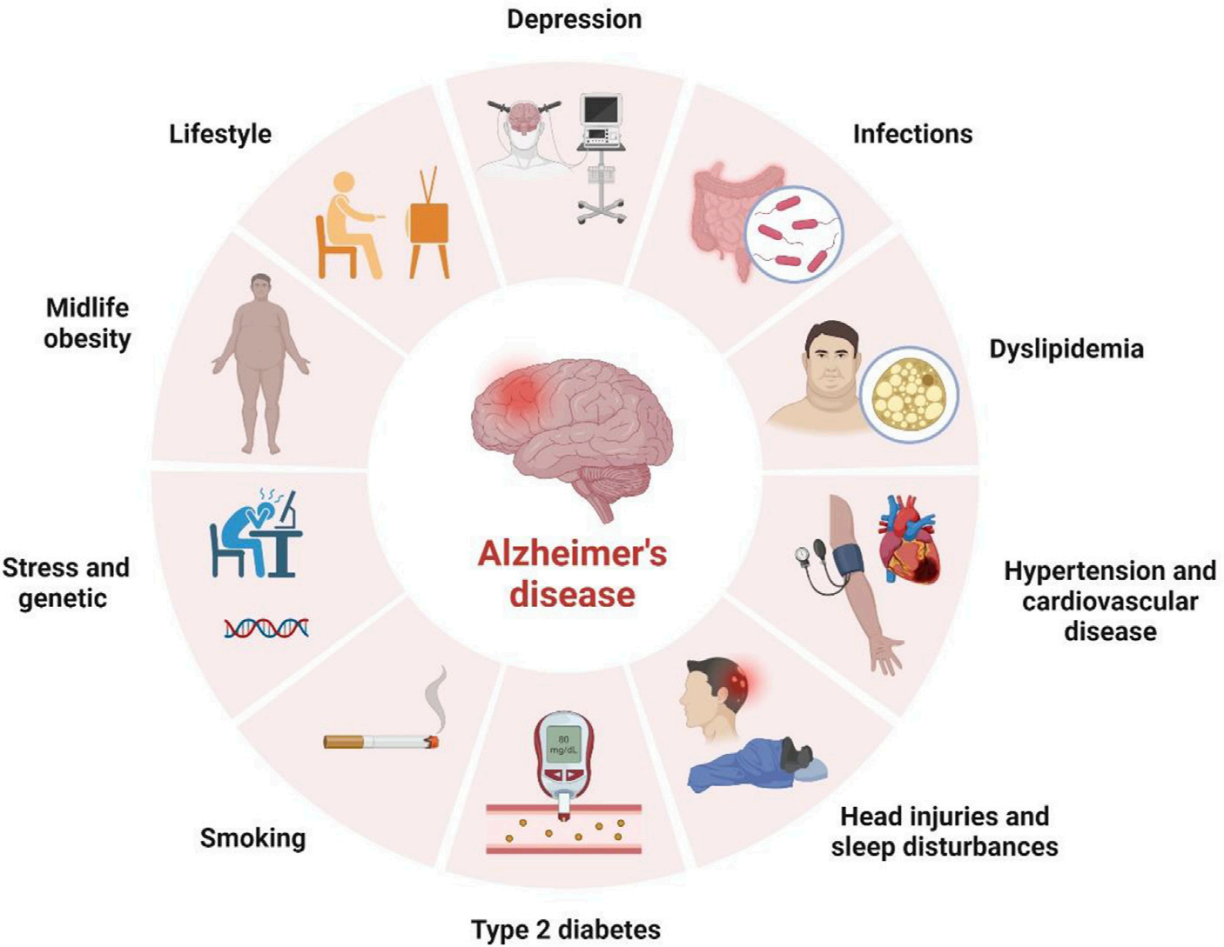
Risk factors of Alzheimer’s Disease.

AD, an essential type of dementia, mainly affects various brain functions like behavior, thinking, and memory. These symptoms, in due course, increase the worsening of daily activities. Memory is lost when 1) crucial meetings are forgotten, 2) familiar tasks such as cooking, driving, writing, and speaking are disoriented, and 3) varied mood swings and withdrawal from relations and family happens (Breijyeh and Karaman, 2020). In addition, many risk factors are correlated with AD, making it a multifactorial disease (Figure 2).

Autophagy, defined as “self-eating,” is a self and systematic degradable process occurring within the cell for recycling cellular components like protein aggregates, misfolded proteins, and unwanted cell organelles (Glick et al., 2010; Fornai and Puglisi-Allegra, 2021). Recent studies on AD mainly focus on autophagy’s contribution to its pathology (Leidal et al., 2018; Aman et al., 2021; Pang et al., 2022). Like other cells, neurons can gather toxic substances/organelles during senescence and require autophagy activation to maintain cell homeostasis (Mariño et al., 2011).

**FIGURE 3:**
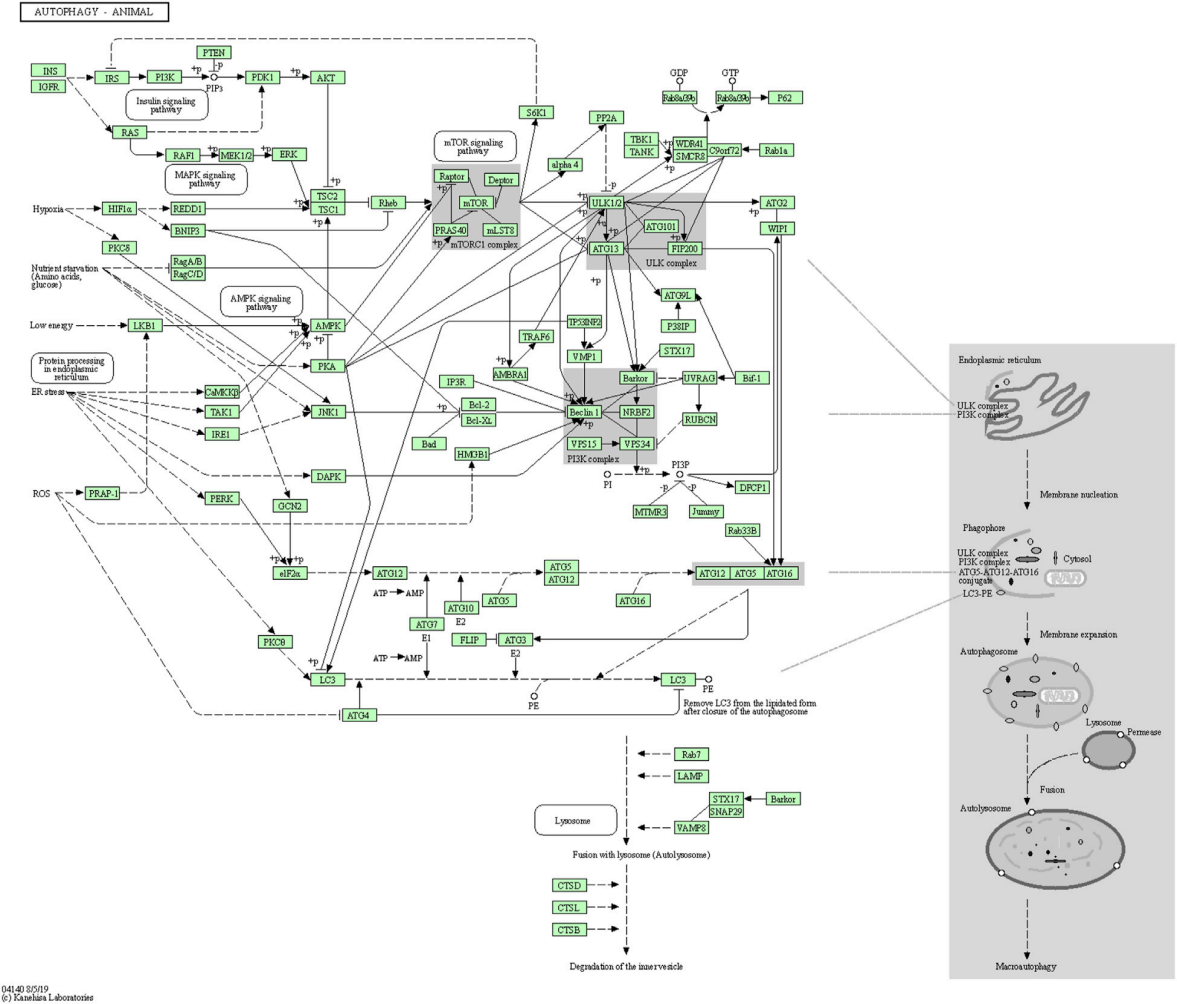
Autophagy pathway (KEGG Id: hsa04140), highlighting its association with the mTOR signaling pathway.

A recent study reported that autophagy-related genes, namely ATG18, ATG8a, and ATG1 in *Drosophila melanogaster* insect model, are down-regulated with age and neuron dysfunction (Zhang et al., 2013). The autophagic flux represents the complete dynamic steps of autophagy including autophagosome formation, maturation, fusion with lysosomes, and subsequent breakdown, followed by the release of macromolecules back into the cytosol (Klionsky, 2008). The autophagy pathway includes releasing various factors, proteins, and signaling molecules. It is associated with other signaling pathways like adenosine monophosphate protein kinase (AMPK), Mammalian target of rapamycin (mTOR), and insulin signaling. The pictorial representation of the autophagy pathway in *Homo sapiens* retrieved from the Kyoto Encyclopedia of Genes and Genomes (KEGG) pathway database is shown in Figure 3 (Kanehisa, 2000).

AD worsens with aging, expressing its symptoms and affecting many brain functions, among which cognitive dysfunction (loss of synapses) and memory loss are prominent (Scheltens et al., 2016). In AD, extracellular senile plaques have amyloid beta particles and intraneural NFT, constituting the major part of aggregated MAPT/Tau protein (Loera-Valencia et al., 2019). The focus on the defects of autophagic flux in AD will not only update the current monitoring methods but also provide visions for developing autophagy-related therapeutics for treating the disease. Through the mTOR signaling, beta-amyloid (Aβ) peptides are accumulated by altering APP metabolism and upregulating β and γ secretases while the mTOR inhibits autophagy function. Moreover, dysregulation of mTOR is allied with many human diseases, like cancer and neurological and metabolic diseases (Chong et al., 2010; Dazert and Hall, 2011; Meng et al., 2013), with more focus now shown on mTOR’s role in the AD’s pathology.

The mTOR is a member of the phosphoinositide-3-kinase-related family with conserved Ser/Thr protein kinase and can respond to environmental stimuli like nutrient concentration, energy state, and growth factors (Yoon, 2017). mTOR is significant for cell growth, metabolism, proliferation, protein translation, and autophagy. Several studies on AD pathology are supported by ample evidence, stressing the association between AD and mTOR signaling (Wang et al., 2014a). The present review is on the divergent mechanistic regulations of autophagy and mTOR proteins, the available inhibitors for mTOR, and how all these combined factors apply to AD’s treatment.

## 2 The role of autophagy in Alzheimer’s disease

**FIGURE 4:**
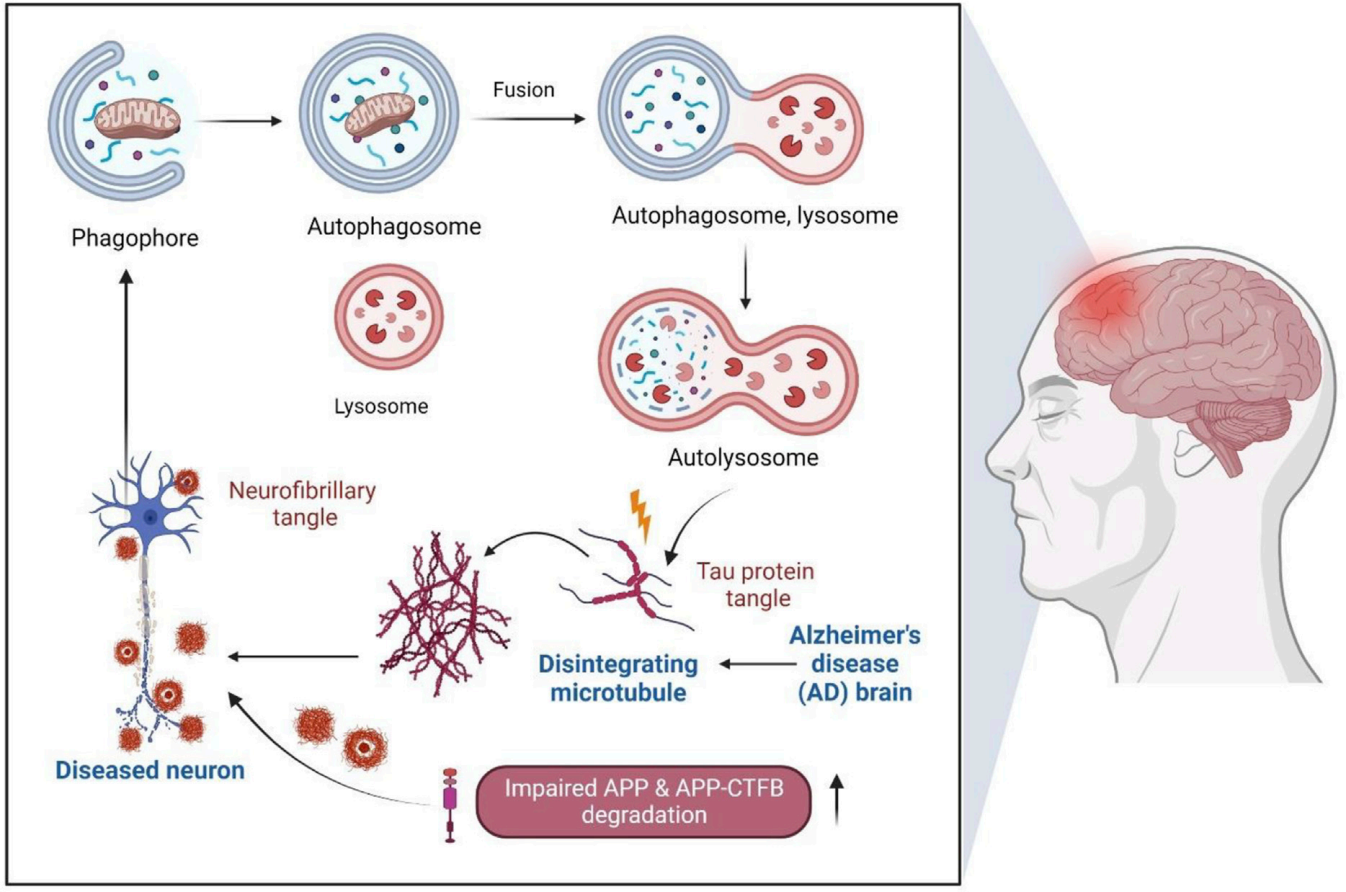
Dysfunction of autophagy-lysosome pathway in AD

Autophagy is a highly conserved pathway for degrading long-lived intracellular proteins, protein aggregates, and organelles (e.g., mitochondrial) *via* lysosomes to maintain homeostasis under physiological conditions (Dikic and Elazar, 2018). Many studies have reported that altered autophagy is directly related to multiple chronic diseases, including AD. Inducing autophagy may therefore result in the removal of Aβ accumulations (Zhang et al., 2022) providing a beneficial effect in preclinical AD models, indicating that autophagy is a reliable tool for developing therapeutic compounds for AD treatment (Figure 4). Moreover, chaperone-mediated autophagy and mitophagy have also been associated with AD (Cuervo and Wong, 2014; Kerr et al., 2017).

The body of scientific evidence suggests the impact of defective mitophagy in the aggregation of faulty autophagosomal vacuoles. Calcium ion imbalance, altered pH, and increased oxidative stress play an important role in this faulty mitochondrial dysfunction mechanism leading to AD progression (Nixon et al., 2008; Nixon, 2013; Medina et al., 2015). Ashrafi et al. (2015) claimed the involvement of PINK1 and PARK2 genes in mitochondrial health maintenance. Mutations occurring in these genes may result in an impaired mitophagy process leading to the clustering of defective autophagosomal vacuoles (Ashrafi and Schwarz, 2015). Another study proposed that mutations in the PSEN1 gene might induce an altered autophagy/mitophagy pathway. This mutation can lead to reduced lysosomal hydrolase activity accelerating the lysosome alkalization resulting in AD (Coffey et al., 2014).

Alteration of the autophagy-lysosome pathway contributes to APP (Torres et al., 2012) and APP-CTFb degradation, overall leading to Aβ aggregate formation. This step also eventually inhibits MAPT/tau aggregates degradation (Inoue et al., 2012) which further induces neurodegeneration. Autophagy is critical in regulating inflammation, where autophagy inducers can also cause glial cells’ autophagy *via* neuron cross-talks (Zhang et al., 2022). Characterization of autophagy-lysosomes impairment in various AD stages, molecules, and genetic types may also pave the way to generate avenues for novel therapeutics.

In another *in vitro* model study, the overexpression of let-7b promoted Aβ1-40 to trigger the phosphatidylinositol-3-kinase (PI3K)/AKT/mTOR pathway in neuroblastoma (SK-N-SH) cells, subsequently inhibiting autophagy and promoting apoptosis (Ji et al., 2006). Some evidence also emphasizes that uncontrolled Aβ accumulations generated more neurotoxicity and progression of AD (Tomiyama et al., 1996). Investigation on Aβ neurotoxicity usually represents SK-N-SH cells and Aβ1-40 as the AD’s cell models (Muñoz and Inestrosa, 1999), thus suggesting that age-induced reduction in autophagy-related gene expression is interlinked with AD in the later stages (Pang et al., 2022). In AD cells, the removal of abnormal protein aggregates can be achieved by utilizing autophagy mechanisms (Metcalf et al., 2012).

Lysosomal acidification and the dysregulation of the V-ATPase complex are common metabolic interruptions associated with AD (Ward et al., 2016) (Colacurcio and Nixon, 2016). But, recent study findings reveal the defective acidification of autolysosomes may induce faulty autophagic build-up of Aβ in neurons (Lee et al., 2022). Therefore, autophagy-stimulating agents/drugs, like mTOR inhibition, remain a potential therapeutic agent for AD without altering the autophagy-lysosomal pathway.

## 3 Dysregulation of mTOR pathway in AD conditions

mTOR is a 289-kD Ser/Thr multidomain protein with an FKBP12 binding and kinase domain which controls many physiological processes. mTOR coordinates the upstream signaling components such as glycogen synthase kinase 3 (GSK-3), growth factors, insulin, AMPK, and PI-3K/Akt (Ferrer et al., 2002; Griffin et al., 2005; Kaper et al., 2006; Gouras, 2013). AD pathogenesis depends on both the down- and upstream regions of mTOR signaling (Cai et al., 2015). Several research studies have highlighted the dysregulation of the mTOR pathway in other diseases like cancer and diabetes (Hsieh and Edlind, 2014) (Habib and Liang, 2014), cardiovascular disease (Chong et al., 2011; Yang and Ming, 2012), aging (Gharibi et al., 2014; Yang et al., 2014), neurodegenerative diseases (Jiang et al., 2013; Sarkar, 2013) as well as obesity (Martínez-Martínez et al., 2014). In fact, some reports stated that mTOR activation contributes to AD progression and interferes with the clinical manifestation and AD pathology (Paccalin et al., 2006; Ma et al., 2010).

Hyperactivated mTOR, the able cause of AD, is regulated with various upstream signaling cascades like GSK3, AMPK (PI3-K)/Akt, and IGF-1. It is also observed that many diseases like mitochondrial dysfunction, auto-immunity, and cancer affect these pathways, causing uncontrolled stimulation of mTOR and leading to tau protein hyperphosphorylation. The phenomenon leads to the formation of NFTs and paired helical filaments (PHFs), the characteristic symptom of AD. Additionally, Aβ plaques are also formed due to the direct inhibition of autophagy by mTOR activation, which induces tau protein hyperphosphorylation and mTOR activities, thus enhancing the advancement of AD (Mueed et al., 2019).

**FIGURE 5:**
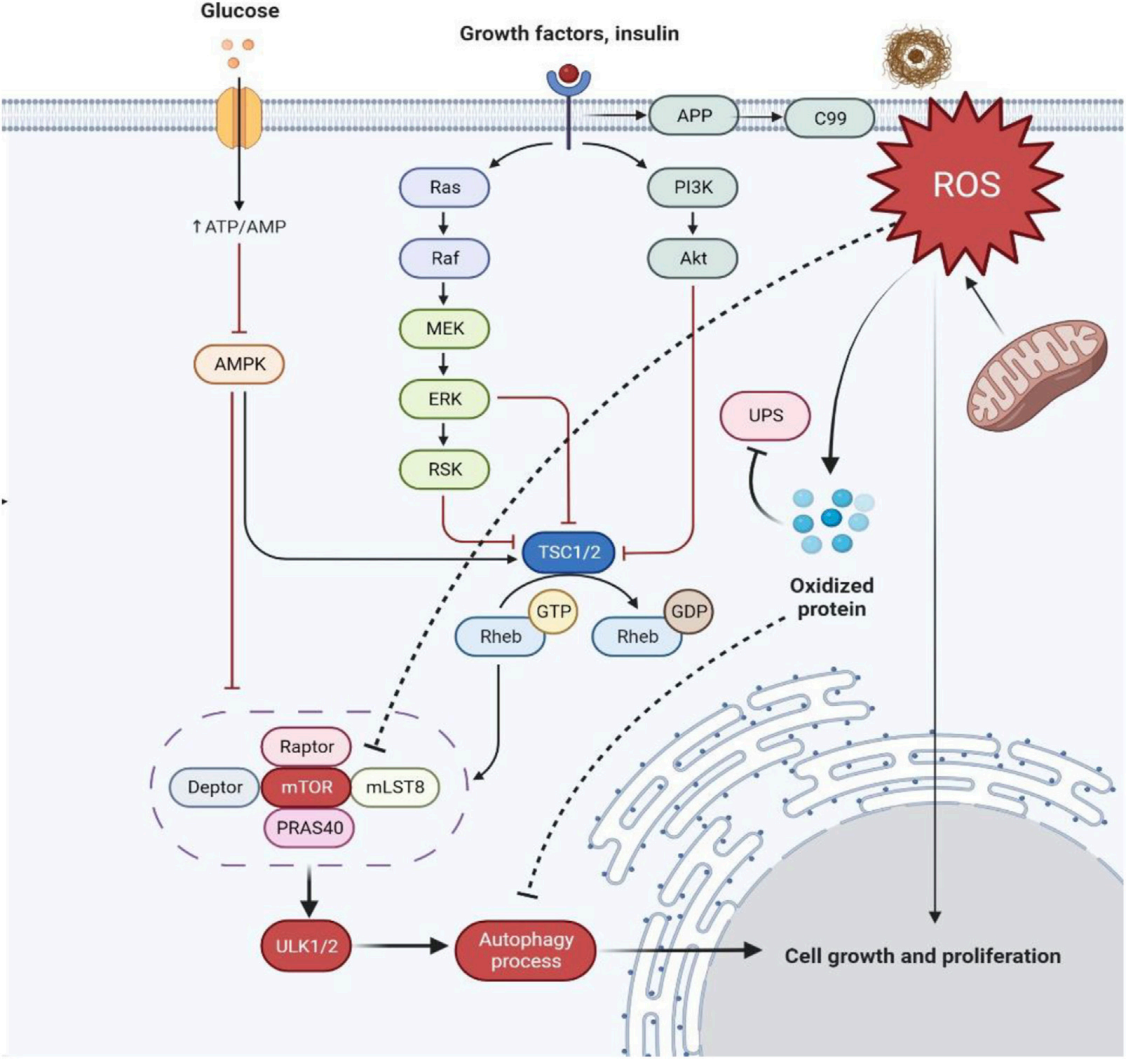
Schematic representation of hyperactivation of mTOR in AD dysregulating insulin signaling and producing more oxidized proteins. Abbreviations: APP, Amyloid precursor protein; ROS, Reactive oxygen species; UPS, Ubiquitin-proteasome system; P13K, Phosphatidylinositol 3-kinase; Akt, Ak strain transforming; Ras, Rat sarcoma; Raf, Rapidly accelerated fibrosarcoma; MEK, Mitogen-activated protein kinase; ERK, Extracellular signal-regulated kinase; RSK, Ribosomal S6 kinase; TSC1/2, Tuberous sclerosis proteins 1/2; GTP, Rheb, Ras homolog enriched in the brain; GDP, Guanosine diphosphate; mTOR, Mammalian target of rapamycin; mLST8, Mammalian lethal with SEC13 protein eight; PRAS40, Proline-rich AKT substrate of 40 kDa; ULK1/2, Unc-51 like autophagy activating kinase; AMPK, AMP-activated protein kinase; ATP/AMP, Adenosine triphosphate/adenosine monophosphate.

Furthermore, it was reported that mitochondrial and nuclear DNA oxidation in AD brains occur with increased levels of 8-oxo-2-dehydroguaninie, 5-hydroxyuracil, and 8-hydroxyadenine in temporal, frontal, and parietal lobes of AD brains (Santos et al., 2012; Perluigi et al., 2021). Likewise, in hippocampus regions of AD brains, heavy levels of 8-hydroxyguanine were also reported (Lovell and Markesbery, 2007; Siman et al., 2015) investigated the selective expression and toxicity of diseased tau by a viral vector approach in the mouse lateral perforant pathway to understanding the activity of rapamycin and its neuroprotective effect. Rapamycin was found to simultaneously inhibit mTOR protein kinase and stimulates autophagy (Siman et al., 2015). The study’s qualitative and quantitative histological findings and morphometric methods revealed a significant reduction in the disease’s symptomatic effects of tau in the perforant pathway upon chronic systematic rapamycin treatment. Henceforth, the progression in the early stages of AD can be addressed by lowering the tau toxicity effect (Figure 5).

Under controlled conditions, decreased levels of free radical or reactive oxygen species (ROS) and Aβ aggregates coordinate stress responses like ubiquitin protease system (UPS), autophagy, and unfolded protein response (UPR) and remove damaged cell organelles and other compounds. Under diseased conditions, ROS are overproduced for the control of protein quality leading to the formation of more oxidized proteins because of protein dysfunction and also leading to the dysregulation of insulin signaling (Tramutola et al., 2015; Höhn et al., 2020).

## 4 Trilateral association between autophagy, mTOR signaling, and Alzheimer's disease

During the persistent circumstance of AD pathology, the mechanistic target of rapamycin complex (mTORC1) regulation is lost, resulting in aggregate formation inside the cell. Indeed, the levels of eukaryotic Initiative Factor 4E (eIF4E) (Li et al., 2005), phosphorylated eukaryotic translation initiation factor 4E-binding protein 1 (4EBP1) (Li et al., 2005), ribosomal protein S6 kinase beta-1 (p70S6K) (Sun et al., 2013), Akt activation (Griffin et al., 2005) and mTOR phosphorylation (at Ser248) are considerably increased in AD-affected brains. These alterations correlate with Braak staging and tau pathology resulting in protein translation disorder. The cognitive decline during AD and mTOR hyperactivation co-exists (Caccamo et al., 2010; Sun et al., 2013). In addition, the levels of phosphate and tensin homologue (PTEN) immunoreactive neurons are decreased in the temporal cortex and hippocampus regions of AD-affected brains showing a negative correlation with senile plaque and NFT formations (Griffin et al., 2005).

The PI3K/Akt signaling is attenuated by PTEN, followed by the dephosphorylation of phosphatidylinositol-3,4,5-triphosphate (PIP3), resulting in the Akt signal hyper-activation to trigger the mTORC1 activity further. This phenomenon leads to the inhibition of autophagy, contributing to Aβ clearance. However, the chances of insulin desensitization need to be checked, which further interrupts Akt activation, as reported in the post-mortem AD brain (Shafei et al., 2017).

**FIGURE 6:**
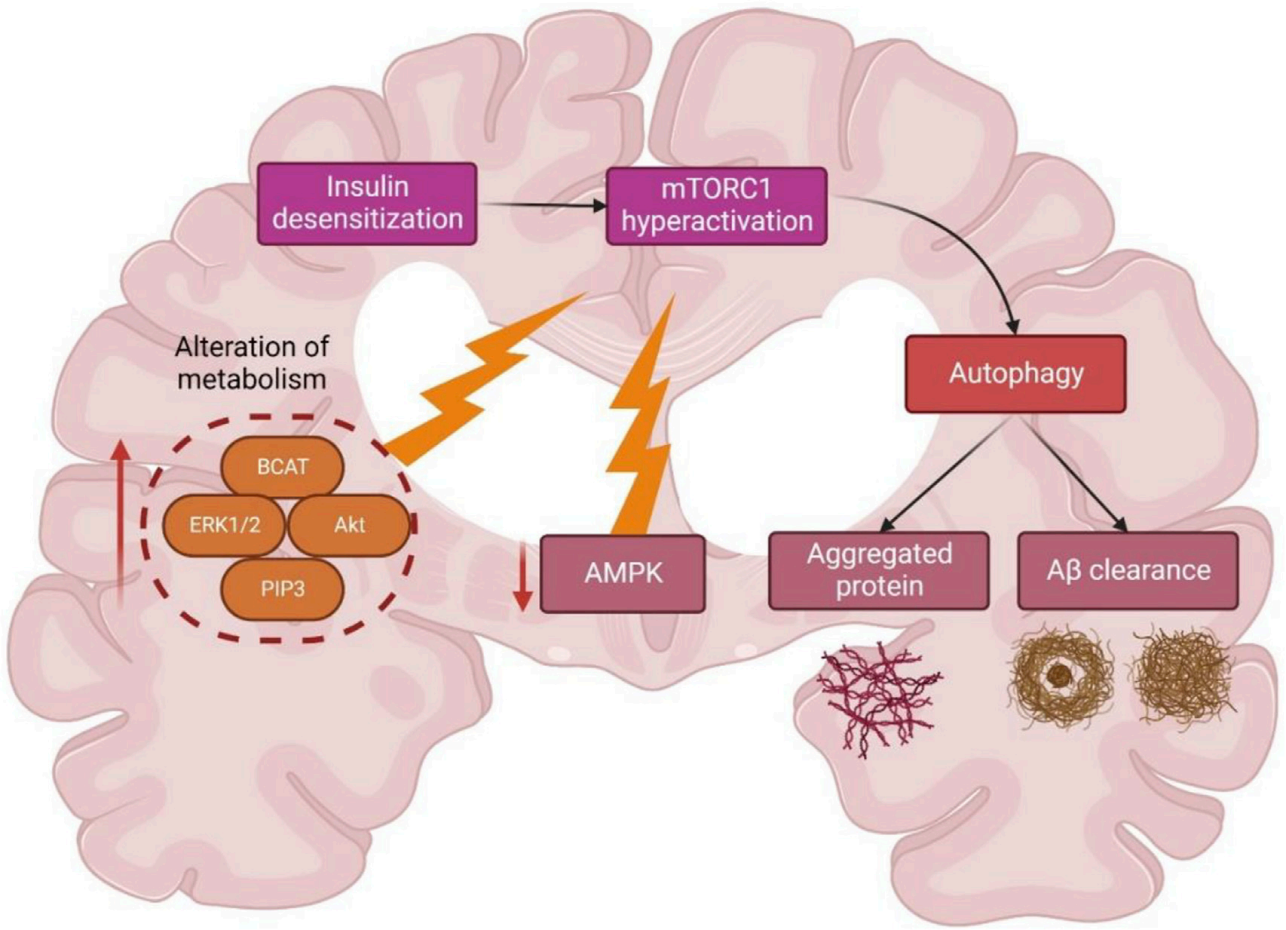
Schematic representation of different metabolic regulations of autophagy and mTOR in Alzheimer’s disease. Abbreviations: BCAT, Branched-chain amino acid aminotransferase; ERK1/2; extracellular signal-regulated kinase 1/2; Akt, Ak strain transforming; PIP3, Phosphatidylinositol (3,4,5)-trisphosphate; AMPK, AMP-activated protein kinase; mTORC1, mammalian target of rapamycin complex 1.

Insulin activation of mTORC1 triggers the extracellular signal-regulated kinases (ERK)_1/2_ pathway, which is upregulated in AD brain and cell models (Morales-Corraliza et al., 2016). During AD, Aβ aggregates worsened mTORC1 signaling due to 40 kDa proline-rich Akt substrate phosphorylation (PRAS40), leading to mTORC1 activity enhancement and autophagy inhibition (Caccamo et al., 2011; Tramutola et al., 2015) (Figure 6). A study (Caccamo et al., 2010) showed the significance of future targets for neuron health regulation by using drug molecules like rapamycin which can inhibit mTOR and induce autophagy by alleviating the accumulation of Aβ peptides. Nevertheless, the mTORC1 upstream region is interlinked with other mechanisms like glucose metabolism, insulin resistance, and AD pathology, thus complicating further studies (Shafei et al., 2017).

In the brains affected with AD, autophagy’s function is altered, leading to the deposition of many toxic proteins. There are many genes, factors, and mechanisms interlinked with the autophagy pathway, which include neuro-inflammation, mTOR signaling, the endocannabinoid system, and UCHL1, UBQLN1, SNCA, PSEN1, MAPT, ITPR1, GFAP, FOXO1, CTSD, CLU, CDK5, BECN1, BCL2, ATG7 signaling pathways (Uddin et al., 2018).

Many autophagy inducers or mTOR inhibitors are used for AD treatment. However, there are several autophagy-inducing agents which require validation. For instance, primary mTORC1 inhibitors termed rapalogs are effective in AD models, while secondary compounds like torins have not been verified. The mTORC1 activity will be blocked by these molecules, like ATP-competitive kinase inhibitors (Zheng and Jiang, 2015). The alteration of cell signaling concerning the mTOR pathway may protect neurons and pose a new approach for neurodegenerative disorders.

Most molecules like calpeptin, minoxidil, and BH3 mimetics, which are autophagy-independent and mTOR-dependent, were not examined in AD models (Sarkar, 2013) (Levine et al., 2015) (Pierzynowska et al., 2018). Henceforward, the studies on the mechanism of autophagy induction by these molecules and the side effects are becoming more relevant for AD treatment (Schmukler and Pinkas-Kramarski, 2020).

### 4.1 Drugs that can suppress/inhibit mTOR

AD has become a globally reported disease with 24 million victims, and the cases are expected to quadruple in due course over the years. Despite the worldwide prevalence, only two types of drug molecules, namely N-methyl d-aspartate (NMDA) antagonists and cholinesterase enzyme inhibitors, have been approved for AD treatment.

Treatment strategies for AD include the use of symptomatic agents such as cholinesterase inhibitors (e.g., donepezil and rivastigmine), disease-modifying therapeutics (e.g., aducanumab and gantenerumab), disease-modifying agents (e.g., lithium and riluzole), chaperone proteins (HSPs), vacuolar sorting protein 35 and several other extracts from natural products (Breijyeh and Karaman, 2020). Many drugs are designed with the function of activating autophagic flux and inhibiting mTOR signaling for AD diagnosis. In recent years, many studies revealed that rapamycin has neuroprotective activity and can act as a pro-autophagy molecule in animal and cell models (Kaeberlein and Galvan, 2019). The toxic nature of various amyloidogenic peptides responds to rapamycin treatment in neuron cell cultures (Boland et al., 2008; Spilman et al., 2010). Similar positive results were observed in other animal models with parameters like aging, protein misfolding, neurotoxicity, and inheritance leading to neurodegeneration (Malagelada et al., 2010).

Several studies on amyotrophic lateral sclerosis (ALS)-affected patients with lithium conveyed positive results in clinical trials (Fornai et al., 2008), and also, recently, the efficiency of rapamycin in AD-affected patients was validated in randomized placebo-controlled Phase-II clinical trials (Mandrioli et al., 2018). Autophagy, i.e., autophagic flux and its modulation, needs 1) complicated and multifaceted signaling, 2) integration coupling of environmental conditions, and 3) functional cell communications that include differentiation, proliferation, and cytoplasmic homeostasis (Thellung et al., 2019).

Rapamycin, a member of the macrolide class, and its analogs, generally known as rapalogs, are meant for the inhibition of mTOR signaling. The said first-generation mTOR inhibitors like everolimus, rapamycin and temsirolimus bind to the 12-kDa FK506 binding protein (FKBP-12) outside the ATP binding pocket and inhibit the mTORC1 kinase activity, keeping mTORC2 unaltered (Ballou and Lin, 2008).

The drugs acting as autophagy activators can be a novel approach for neural protection by reducing the toxicity levels of misfolded proteins (Thellung et al., 2019). In general, all neurodegenerative disorders show similar pathogenic mechanisms like autophagic flux impairment losing the ability to degrade the neurotoxic oligomers of wrongly folded proteins. Nevertheless, the autophagy process can be pharmacologically activated by hindering the enzymatic activity of mTORC1. Consequently, its autophagy suppressing activity, found in the physiological condition, is lost. Similarly, rapamycin is the first drug that acts pharmacologically by enhancing autophagy inducing neuroprotection, and offering the clearance of oligomers. This step necessitates disease-modifying strategies to trigger the development of new compounds and also the modification of existing drug molecules for better pro-autophagic potentiality.

**FIGURE 7:**
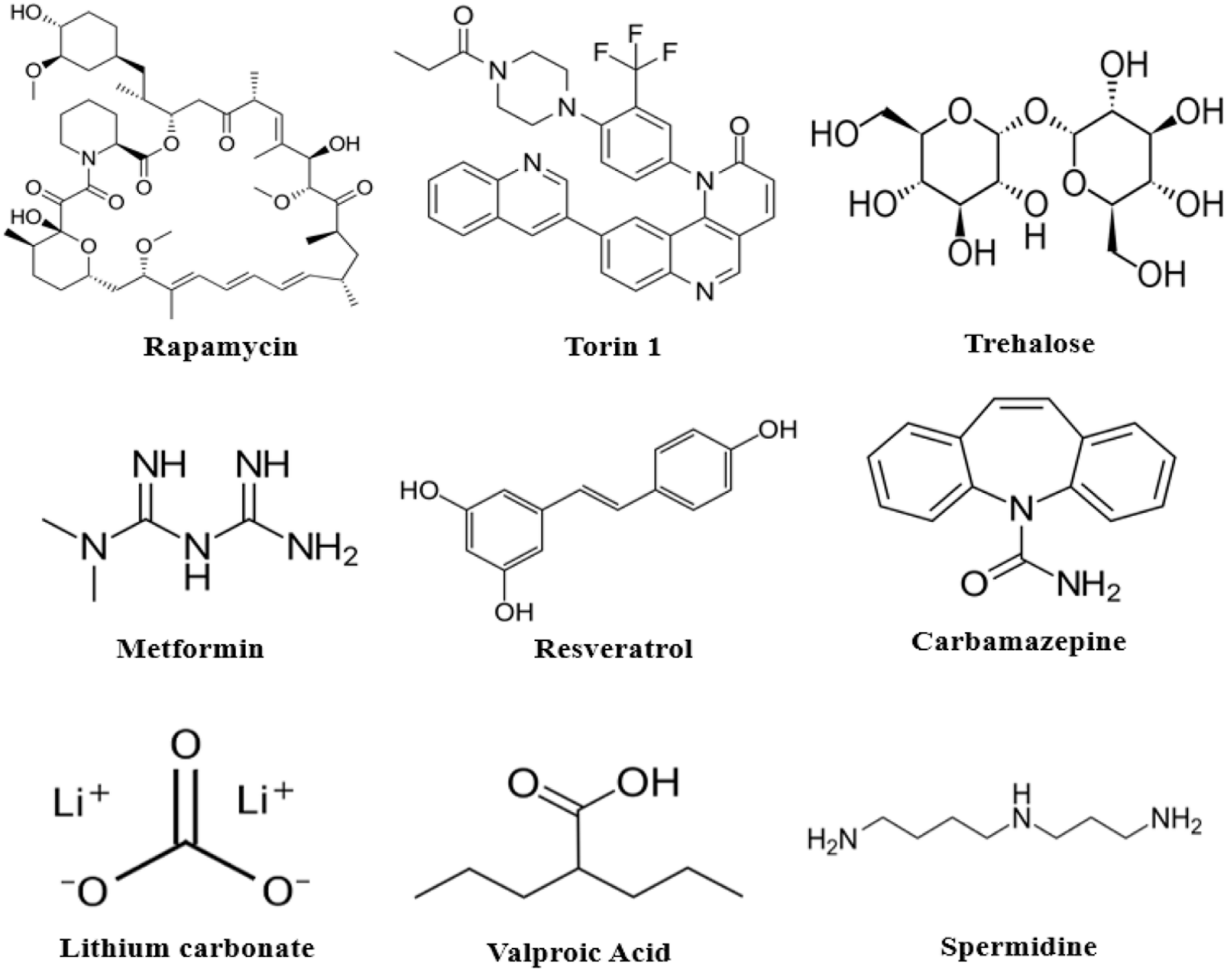
Chemical structures of some autophagy-inducing agents.

The rapalogs and rapamycin act as autophagy inducers by stabilizing raptor-mTOR connectivity and inhibiting mTOR stimulation (Lamming et al., 2013). Torin1 and dactolisib can also inhibit mTOR signaling. Other compounds that activate AMPK signaling, like trehalose, metformin, and resveratrol, promote mTOR inactivation and are AMPK-dependent (Figure 7). Furthermore, compounds such as Apelins (Jiang et al., 2020), Tramadol (Soltani et al., 2020), Curcumin (Wang et al., 2014b; Zhu and Bu, 2017), Cubebene (Li et al., 2019), Gemfibrozil (Luo et al., 2020), β-asarone (Wang et al., 2020) and Salidroside (Rong et al., 2020) promote inhibition of mTOR *via* inhibition of phosphoinositide/Akt pathway.

### 4.2 Validation of the role of autophagy induction in Alzheimer’s disease: Concepts and queries

One of the key considerations in assessing the effect of autophagy induction on AD prevention is that the chemical molecules are non-specific and, therefore, may affect other processes in the cell. Henceforth, to verify and understand the effects and treatment modes of the molecules, the autophagy-inducing agent enhancing autophagy in AD must be demonstrated, on which the therapeutic effect of the said molecule relies on.

Table 1 lists several autophagy inducers and their mechanism of autophagy induction, which acts *via* the direct or indirect inhibition of mTOR (Heras-Sandoval et al., 2020). Few other drugs/molecules were also claimed to induce autophagy, but they may trigger defective autophagy process by modifying the autophagosome formation, autophagosome maturation, and the autophagy-lysosomal pathway *via* faulty lysosomal acidification (Uddin et al., 2019). Another critical challenge is whether the autophagy inducers virtually influence all AD types and whether it must be applied to populations based on the disease stage and the patient genetic history.

**TABLE 1:** List of some Autophagy inducers/activators that may be useful in AD.

Autophagy inducer	Chemical formula	Inducing pathway	Clinical sign/Nature of the molecule
Rapamycin	C_51_H_79_NO_13_	Direct inhibition of mTORC1	Immunosuppressant, anti-fungal, anti-cancer
Metformin	C_4_H_11_N_5_	Activation of AMPK	Anti-diabetic
Torin1	C_35_H_28_F_3_N_5_O_2_	Blockade of ATP binding site on mTOR	Anti-cancer
Trehalose	C_12_H_22_O_11_	Activation of AMPK	Antioxidant
Resveratrol	C_14_H_12_O_3_	Activation of AMPK; upregulation of ATGs expression	Antioxidant
Lithium carbonate	Li_2_CO_3_	Inhibition of GSK-3β; Inhibition of the phosphoinositide cycle	Mood stabilizer
Valproic acid	C_8_H_16_O_2_	Inhibition of the phosphoinositide cycle; inhibition of GSK-3β	Anti-epileptic, mood stabilizer
Spermidine	C_7_H_19_N_3_	Prevention of beclin 1 cleavage; upregulation of ATGs expression; inhibition of EP300	Natural polyamine
Carbamazepine	C_15_H_12_N_2_O	Inhibition of phosphoinositide cycle; inhibition of GSK-3β	Analgesic, anti-epileptic, mood stabilizer
Apelin	C_266_H_427_N_95_O_71_S_2_	Inhibition of the phosphoinositide cycle	Peptide
Tramadol	C_16_H_25_NO_2_	Inhibition of the phosphoinositide cycle	Opioid analgesic
Gemfibrozil	C_15_H_22_O_3_	Inhibition of the phosphoinositide cycle	Fibrate
Salidroside	C_14_H_20_O_7_	Activation of AMPK	Natural glycoside
β-asarone	C_12_H_16_O_3_	Akt—mTOR inhibition	Natural phenylpropanoid
Curcumin	C_21_H_20_O_6_	Inhibition of phosphoinositide/Akt pathway	Antioxidant, Natural polyphenol
Cubebene	C_15_H_24_	Inhibition of phosphoinositide/Akt pathway	Natural sesquiterpene

Overall, since the combined hypothesis on molecular mechanisms of neuron destruction in various neurodegenerative disorders includes autophagy pathway malfunction leading to oligomer aggregation, analysis of the utilization of pro-autophagic drugs will validate this role further, even in AD conditions. Specifically, existing and novel compounds are under preclinical and clinical trials for the induction of autophagy through oligomer clearance. Besides, many existing drugs will have off-target effects. Hence, there remains the necessity for developing novel assays for detecting autophagic flux in clinical trials of animal and human models.

## 5 Conclusion

Despite the collection of huge data in the current review on the role of autophagy in AD treatment, the proper functioning of autophagy and the mechanical induction of autophagy by drug compounds is decisive for aging and neurons in a natural way. The defect in the autophagic mechanism of neurons is one of the projecting factors that generate neurodegenerative disorders like AD. Although its therapeutic effect requires further validation studies, and since autophagy is mainly affected in AD, autophagy remains a primary innovation as a new therapeutic target in the form of autophagy inducers.

The Aβ and tau protein metabolism pathways, mTOR signaling, and autophagy’s role are significant, including mediating effects in neuro-inflammation and endo-cannabinoid systems, and are tremendously influenced by the autophagy process acting as intermediating agents during AD conditions. Therefore, therapeutic approaches targeting the autophagy mechanism would pave the way for expanding novel strategies for AD management. The new approaches should include therapeutics that inhibit mTOR signaling and induce autophagy without altering the other interconnected mechanisms.

## Glossary

AD: Alzheimer’s Disease
APP: Amyloid Precursor Protein
NFTs: Neurofibrillary Tangles
mTOR: Mechanistic Target of Rapamycin
mTORC1: Mechanistic Target of Rapamycin Complex
UCHL-1: Ubiquitin C-Terminal hydrolase L1
UBQLN1: Ubiquitin-1
SNCA: Alpha Synuclein gene
PSEN1: Presenilin-1
MAPT: Microtubule Associated Protein tau
ITPR1: Inositol 1,4,5-triphosphate receptor type-I
GFAP: Glial Fibrillary Acidic Protein
FOXO1: Forkhead Box Protein O1
CTSD: Cathepsin D
CLU: Clusterin
CDK5: Cyclin dependent kinase 5
BECN1: Beclin-1
BCL 2: B-Cell lymphoma 2
ATGs: Autophagy-related genes
AMPK: AMP-activated Protein Kinase
APPCTF: C-terminal Fragments of Amyloid Precursor Protein
PI3K: Phosphatidyl Inositol 3 Kinase
AKT: Serine/Threonine kinase
PHFs: Paired Helical Filament
ROS: Reactive Oxygen Species
UPS: Ubiquitin Protease System
UPR: Unfolded Protein Response
EIF4E: Eukaryotic Initiative Factor 4E
Ebp1: ErbB3-Binding Protein
P70s6k: Ribosomal Protein S6 Kinase beta-1
PTEN: Phosphate and Tensin Homologue
FKBP: FK506 Binding Protein
ATP: Adenosine Triphosphate
GSK3β: Glycogen Synthase Kinase 3 Beta
PARK2: Parkin 2
PINK1: PTEN induced putative kinase 1
